# Long-Term IGF-I Exposure Decreases Autophagy and Cell Viability

**DOI:** 10.1371/journal.pone.0012592

**Published:** 2010-09-07

**Authors:** Alessandro Bitto, Chad Lerner, Claudio Torres, Michaela Roell, Marco Malaguti, Viviana Perez, Antonello Lorenzini, Silvana Hrelia, Yuji Ikeno, Michelle Elizabeth Matzko, Roger McCarter, Christian Sell

**Affiliations:** 1 Department of Pathology, Drexel University College of Medicine, Philadelphia, Pennsylvania, United States of America; 2 Department of Biochemistry, “G. Moruzzi” University of Bologna, Bologna, Italy; 3 Barshop Institute for Longevity and Aging Studies, University of Texas Health Science Center at San Antonio, San Antonio, Texas, United States of America; 4 Barshop Institute for Longevity and Aging Studies and Department of Pathology, University of Texas Health Science Center at San Antonio, Research Service, Audie Murphy VA Hospital (STVHCS), San Antonio, Texas, United States of America; 5 Department of Biobehavioral Health, Penn State University, State College, Pennsylvania, United States of America; University of Washington, United States of America

## Abstract

A reduction in IGF-I signaling has been found to increase lifespan in multiple organisms despite the fact that IGF-I is a trophic factor for many cell types and has been found to have protective effects against multiple forms of damage in acute settings. The increase in longevity seen in response to reduced IGF-I signaling suggests that there may be differences between the acute and chronic impact of IGF-I signaling. We have examined the possibility that long-term stimulation with IGF-I may have a negative impact at the cellular level using quiescent human fibroblasts. We find that fibroblast cells exposed to IGF-I for 14 days have reduced long-term viability as judged by colony forming assays, which is accompanied by an accumulation of senescent cells. In addition we observe an accumulation of cells with depolarized mitochondria and a reduction in autophagy in the long-term IGF-I treated cultures. An examination of mice with reduced IGF-I levels reveals evidence of enhanced autophagy and fibroblast cells derived from these mice have a larger mitochondrial mass relative to controls indicating that changes in mitochondrial turnover occurs in animals with reduced IGF-I. The results indicate that chronic IGF-I stimulation leads to mitochondrial dysfunction and reduced cell viability.

## Introduction

A major paradox exists in the current understanding of how insulin-like growth factors affect the aging process. Mammalian insulin-like growth factor 1 (IGF-I) is critical for cellular proliferation, muscle and adipose tissue differentiation, and neural development [1]–[9]. Moreover, IGF-I enhances cell survival in the face of numerous physiologic insults that include DNA damage [10], [11] and loss of cell adhesion [12]–[14]. The IGF-I paradox lies in the fact that despite the proliferation- and survival-enhancing properties attributed to IGF-I, it is a reduction in IGF-I signaling that has been shown to extend lifespan in multiple organisms including nematodes, flies, and mammals [15]–[21]. The paradoxical effects of IGF-I on cell survival, differentiation, and lifespan suggest that there may be a tradeoff between short-term benefit and long-term survival. However, the molecular mechanisms that underlie this tradeoff remain unclear. It is possible that long-term negative consequences of IGF-I stimulation cannot be fully appreciated in the culture systems presently used to study cell growth, survival, and differentiation. In vivo, these consequences may be difficult to identify due to the pleiotrophic effects of IGF-I.

In order to examine the possible consequences of increased IGF-I signaling over extended periods, a fibroblast culture model was developed that allows human fibroblasts to be maintained in a quiescent state over a period of weeks. This model takes advantage of MCDB 105 culture medium, which has been specifically formulated for survival and growth of human fibroblasts in low serum [22]. The medium provides all essential amino acids, glucose, nutrients, and trace elements required for proliferation. When growth factors are withdrawn, fibroblast cells enter a non-dividing quiescent state that is fully reversible upon the addition of growth factors or serum. Using similar conditions, human fibroblasts cultures have been maintained under serum free conditions for up to 3 weeks without impacting growth potential [23]. We have utilized this in vitro approach to examine cellular responses to unopposed signaling through the IGF-I receptor over a period of weeks. We report here that human fibroblast cultures maintained in MCDB 105 medium responded to IGF-I with a reduction in long-term viability as judged by colony forming assays and an increased percentage of cells expressing senescence-associated beta-galactosidase (SA-β-gal). These changes are accompanied by a reduction in autophagy and the accumulation of a subpopulation of cells that display reduced mitochondrial potential. Using a mouse model with reduced IGF-I production, we find evidence for enhanced autophagy in several tissues including liver and kidney. Furthermore, fibroblast cells cultured from these animals show an increase in mitochondrial mass relative to fibroblasts derived from control animals.

## Results

**Figure 1:**
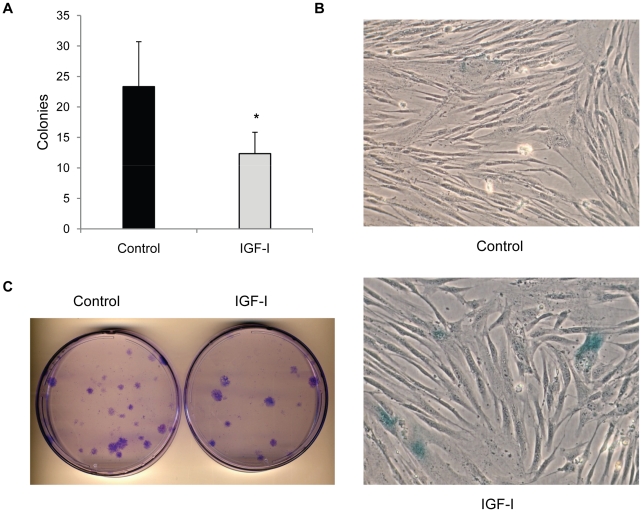
IGF-I decreases long-term viability of human fibroblasts. Long-term IGF-I treatment reduces colony formation potential. Colony forming assays were performed on cells that had been maintained in either MCDB 105 medium without additions or with IGF-I (40 ng/ml) for 2 weeks. Cells were seeded in full growth medium to allow colony growth and results are presented in panel **A**. Bars are number of colonies per 3×10^3^ cells plated (*, *P*<0.01) **B.** Representative micrograph (20X) of senescence-associated β-galactosidase staining of fibroblast colonies. **C.** Crystal violet stained colonies of plates seeded with 3×10^3^ cells for colony forming assays.

In order to examine the consequences of prolonged IGF-I stimulation, viability and clonal growth was examined in cultures exposed to IGF-I for 14 days. Under these conditions, human fibroblasts remain quiescent even in the presence of IGF-I due to the need for additional stimulation with EGF for entry into S phase [24] and no cell loss was observed in any of the conditions used. Colony forming ability was significantly reduced in cultures treated with IGF-I in relation to parallel cultures that were maintained in the nutrient rich MCDB 105 media without growth factors (Fig 1a,c). In addition, IGF-I–treated cultures contained cells that stained positive for SA-β-gal, a marker of replicative senescence, suggesting that cells within the population had been driven into a senescent state during the incubation period (Fig 1b).

**Figure 2:**
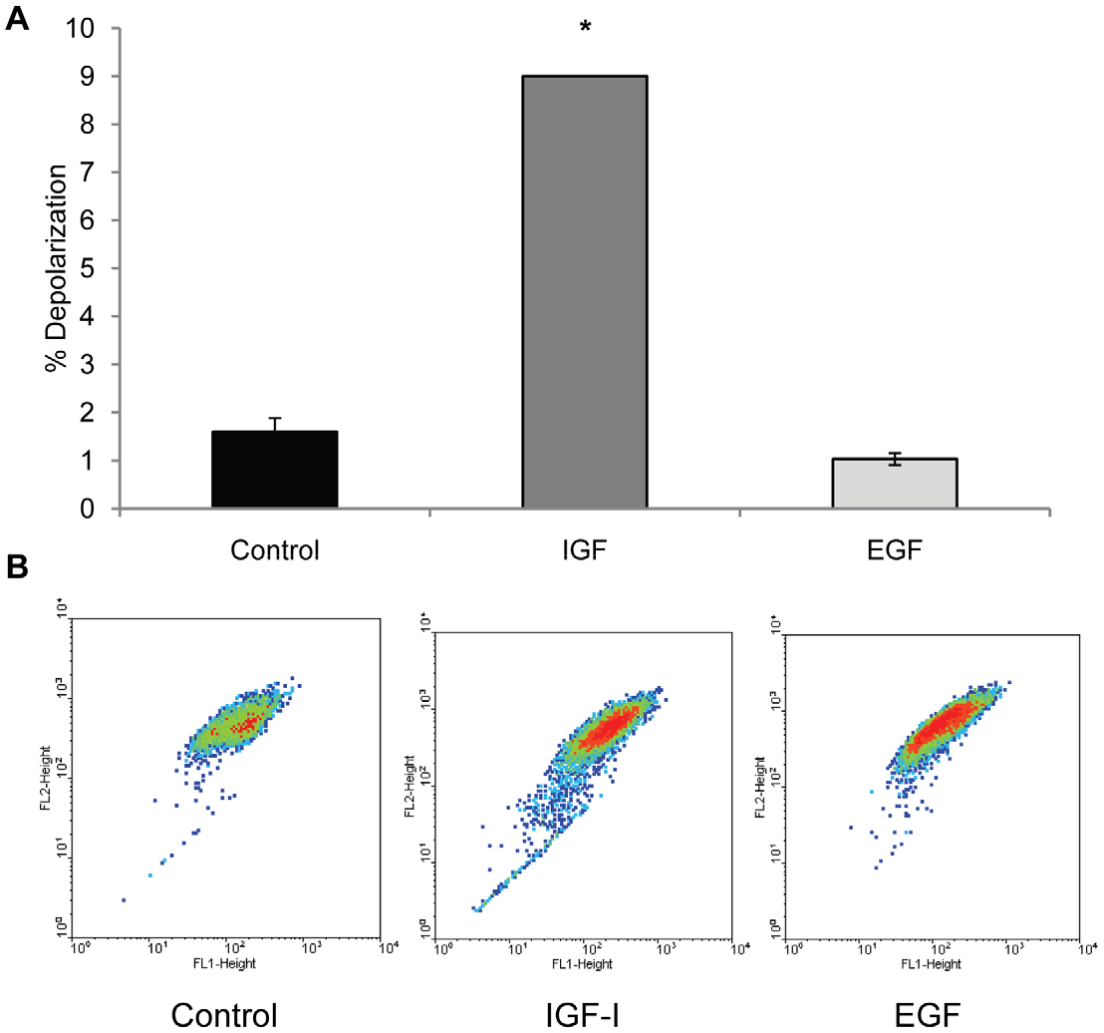
IGF-I treatment increases mitochondrial depolarization. WI-38 fibroblasts were maintained for 14 days in MCDB 105 medium, MCDB 105 medium with IGF-I (40 ng/ml), or MCDB 105 medium with EGF (40 ng/ml). Medium with or without growth factors was replenished every 3 days and cells were stained for mitochondrial potential at that time as described in materials and methods. Cells with depolarized mitochondria were visualized by flow cytometry as described in Material and Methods. **A.** Percentage of cells with depolarized mitochondria as assessed by JC-1 staining (*, *P*<0.001). **B.** Representative dot blot of JC-1-stained cultures in MCDB 105 with or without IGF-I or EGF. Y-axis, fluorescence at 590 nm; X-axis, fluorescence at 525 nm. A downward shift on the X-axis is indicative of mitochondrial membrane depolarization.

During the period of IGF-I exposure we observed unexpected changes such as cellular inclusions and vacuolization, which began to appear in cells after 5-7 days (not shown). Both the number of cells with vacuoles and the number of vacuoles per cell increased with increased time in culture. Parallel cultures that were maintained in serum free medium without IGF-I did not accumulate intracellular vacuoles although it should be noted some intracellular inclusions do appear. Because mitochondria are critical to cellular viability and IGF-I is known to influence mitochondria through several signaling pathways, we postulated that mitochondrial damage may occur with extended exposure to IGF-I. As a marker of mitochondrial integrity, mitochondrial membrane potential was examined using the cationic dye JC-1. The emission spectrum of JC-1 monomers is 525 nm while aggregates that form in the mitochondria due to the membrane potential fluoresce at 590 nm. Cells with a decreased fluorescence of JC-1 aggregates (590 nm), indicative of decreased membrane potential (and dysfunctional mitochondria), accumulated in cultures exposed to IGF-I beginning at 7–10 days in culture up to the maximum time examined, at 14 days (Fig 2). Interestingly, this population of cells was significantly smaller in cultures that were exposed to EGF rather than IGF-I for the same period, indicating that specific signals generated by the IGF-I receptor are responsible for the effect on mitochondrial membrane potential.

**Figure 3:**
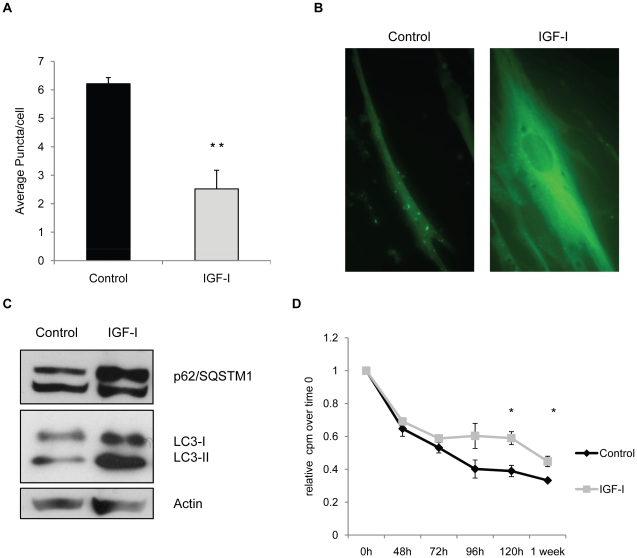
IGF-I treatment impairs autophagy. WI-38 fibroblasts stably expressing the GFP-LC3 fusion protein were maintained for 14 days in MCDB 105 medium, or MCDB 105 medium with IGF-I (40 ng/ml). Medium with or without growth factor was replenished every 3 days. **A.** Number of LC3 puncta per cell in WI-38 GFP-LC3 cells with or without 40 ng/ml IGF-I (**, *P*<0.01) At least 25 fields and 100 cells per slide were examined. **B.** Representative fluorescence micrograph (40X) of WI-38 GFP-LC3 cells with or without IGF-I treatment. **C.** Accumulation of LC3 and p62/SQSTM1 over time in IGF-I-treated cells as assessed by western blot. **D.** Protein degradation in control and IGF-I-treated cells measured as percentage of the residual ^35^S-Methionine radioactivity at the indicated time points over time 0 (*, *P*<0.01).

Under conditions of stress it is thought that intracellular components such as mitochondria are targeted for degradation by autophagy and we postulated that a basal level of autophagy may be required for normal cellular homeostasis in quiescent cultures. Accordingly, we examined the process of autophagy to determine whether it was active in the quiesecent cultures despite the fact that all essential nutrients were provided. In order to visualize the process of autophagy, we introduced a fluorescent-tagged version of the LC-3 protein, a key component of the autophagosome, into human fibroblast cells. The accumulation of GFP-LC3 as intracellular puncta is thought to represent the formation of autophagosomes [25]. Fluorescent puncta could be visualized in fibroblast cells maintained for 14 days in MCDB 105 medium and addition of IGF-I decreased the appearance of these puncta significantly (Fig 3a,b). Interestingly, the IGF-I treated cultures contained very high levels of the GFP-LC3 protein, however, there were few puncta and the GFP-LC3 protein appeared to be associated primarily with the cytoskeleton (Fig 3b). Proteolytic processing to remove a C-terminal portion and conjugation with phosphatidyl ethanolamine is required for LC-3 incorporation into autophagosome membranes and the 2 forms, LC3-I (cytosolic) and LC3-II (membrane bound) can be visualized by Western blot analysis. Fibroblasts incubated in MCDB 105 with or without IGF-I were examined for LC3-I and LC3-II using an antibody that recognizes both forms of the protein. IGF-I treated cells contained higher levels of LC3 (Fig 3c). Over the course of multiple experiments we observed a consistent increase in the levels of LC3-I and LC3-II, suggesting that processing of the protein occurs but that autophagosome formation is suppressed.

To confirm that the changes in LC-3 were indicative of a reduction in autophagy, we examined the levels of p62/A170/SQSTM1, a long lived protein that is degraded through autophagosomes. This protein accumulates when autophagy is inhibited [26]. The levels of p62 progressively increase in IGF-I treated cultures consistent with an inhibition of autophagy (Fig 3c). In addition, we examined the degradation of cellular proteins over a 1-week time course (Fig 3d). Similar to our results with p62, we find that the degradation of long-lived cellular proteins is reduced in the presence of IGF-I consistent with a reduced rate of autophagy.

**Figure 4:**
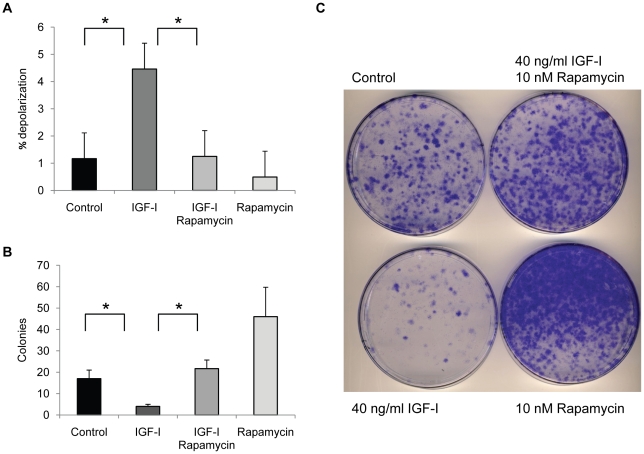
Rapamycin restores mitochondrial clearance and long-term viability. WI-38 fibroblasts were maintained for 14 days in MCDB 105 medium, or MCDB 105 medium with IGF-I (40 ng/ml). Medium with or without growth factor was replenished every 3 days. Mitochondrial potential and colony forming assays were performed at the end of the 14 day period. **A.** Rapamycin reduces the effect of IGF-I on mitochondria depolarization as assessed by JC-1 staining (*, *P*<0.05). **B.** Rapamycin restores long-term viability in IGF-I-treated cells as measured by colony forming assay. Bars are number of colonies per 3×10^3^ cells plated. **C.** Crystal violet stained colonies of plates seeded with 3×10^4^ cells after 2 weeks in full growth medium.

**Figure 5:**
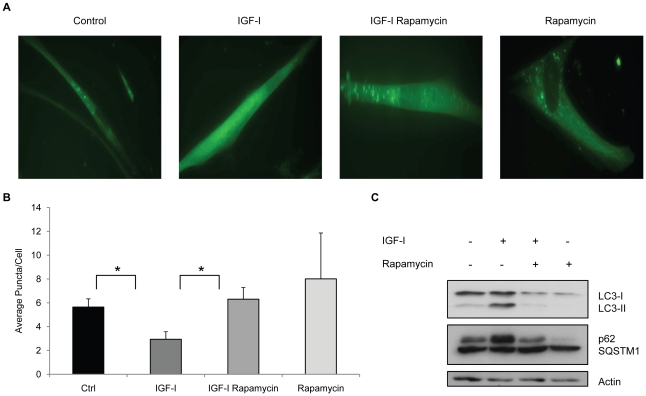
Rapamycin restores autophagy in IGF-I-treated cells. WI-38 fibroblasts stably expressing the GFP-LC3 fusion protein were maintained for 14 days in MCDB 105 medium, or MCDB 105 medium with IGF-I (40 ng/ml). Medium with or without growth factor was replenished every 3 days. **A**. Representative fluorescence micrographs of WI-38 GFP-LC3 cells with or without 40 ng/ml IGF-I and/or 10 nM rapamycin. **B.** Number of LC3 puncta per cell in WI-38 GFP-LC3 cells (*, *P*<0.05). At least 25 fields and 100 cells per slide were examined **C.** Levels of LC3 and p62/SQSTM1 in WI-38 with or without 40 ng/ml IGF-I and/or 10 nM rapamycin as assessed by Western blot analysis.

**Figure 6:**
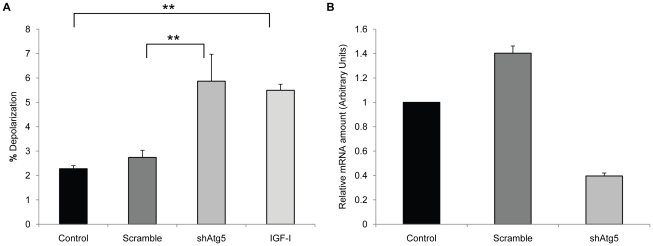
Impairment of autophagy increases mitochondrial depolarization. WI-38 fibroblasts and WI-38 cells expressing either an shRNA construct targeting Atg5 (shAtg5) or the same targeting vector expressing a scrambled sequence (Scramble) were maintained for 14 days in MCDB 105 medium. As a control, parallel cultures were maintained in MCBD 105, or MCDB 105 medium with IGF-I (40 ng/ml). Medium with or without growth factor was replenished every 3 days. Cells were stained for mitochondrial potential at that time as described in materials and methods. Cells with depolarized mitochondria were visualized by flow cytometry as described in Material and Methods. Differences in the percent of cells with depolarized mitochondria between cell populations were significant for shAtg5 versus scramble or control cells (*, *P*<0.05). The difference in the percent of cells with depolarized mitochondria between IGF-I treated and either control or scramble was also significant (*, *P*<0.05).

If the accumulation of depolarized mitochondria was due to changes in autophagy, then one would expect that inducers of autophagy would prevent this accumulation. We tested the effect of rapamycin, a strong inducer of autophagy, in the human fibroblasts treated with IGF-I. Rapamycin prevented the accumulation of cells with depolarized mitochondria induced by IGF-I treatment, as judged by JC-1 staining (Fig. 4a) and restored long-term viability (Fig. 4b,c). Rapamycin treatment alone increased colony forming potential in cultures that did not receive IGF-I suggesting that rapamycin was able to increase viability of cells in serum free conditions although this difference was not statistically significant. In addition, rapamycin restored autophagy in IGF-I treated cells, as judged by the numbers of GFP-LC3 puncta (Fig 5a,b) and the levels of LC3-I and p62 (Fig 5c) Thus, a specific inhibitor of mTOR, an important regulator of autophagy, prevented the negative aspects of long-term IGF-I treatment. An independent assessment of the influence of autophagy on mitochondrial changes was provided by introducing into WI-38 cells an shRNA plasmid vector that targets ATG5, an essential gene for autophagy [27]. Knock down of ATG5 transcript was confirmed using real time PCR analysis of mRNA levels for ATG5 and the cells were placed into long-term quiescence. Consistent with a role for autophagy in mitochondrial clearance, ATG5 knock down produced an increase in the population of cells with depolarized mitochondria after 2 weeks of quiescence (Fig 6).

**Figure 7:**
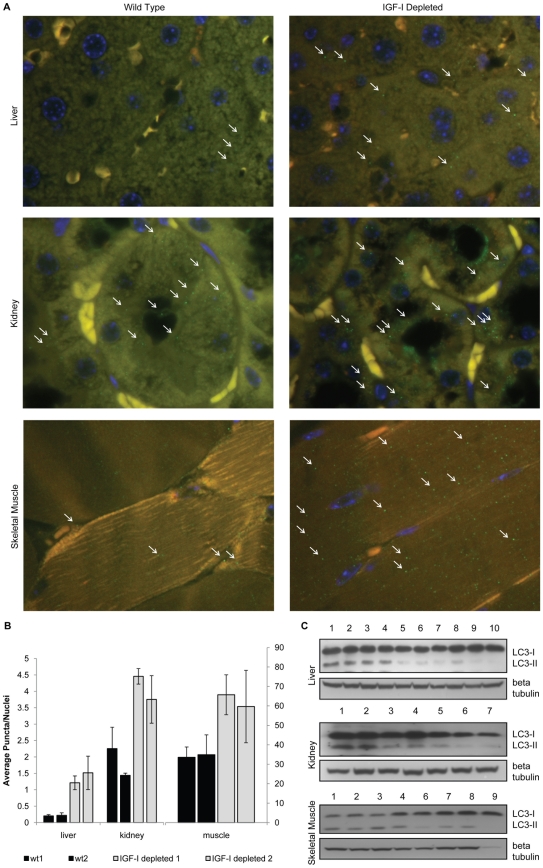
IGF-I-depleted mice show markers of increased autophagy. Tissue sections from IGF-I deficient mice and controls were examined for LC3 containing puncta as described in the Material and Methods section. **A.** Representative fluorescence micrograph of liver, kidney, and quadriceps tissue slides from wild type and IGF-I-depleted mice stained with anti-LC3 rabbit polyclonal antibody. **B.** Number of LC3 puncta per nuclei in tissues from wild type and IGF-I-depleted mice. At least 100 nuclei and 25 fields per slide were examined. **C.** Western blot analysis of LC3 protein levels in liver, kidney, and skeletal muscle tissue lysates. 1,2,3: IGF-I-depleted mice starved for 24 hours. 4,5: IGF-I-depleted mice fed. 6,7,8: wild type mice starved for 24 hour. 9,10: wild type mice fed.

**Figure 8:**
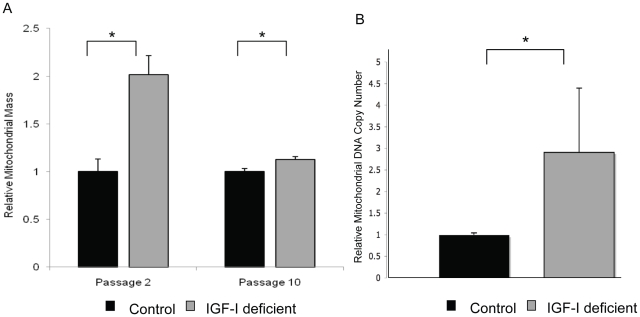
Mouse embryo fibroblasts from IGF-I-depleted mice show increased mitochondrial mass and DNA content. Mouse embryo fibroblasts (MEFs) were growth from IGF-I deficient mice or control animals as described in materials and methods. The relative mitochondria content in wild type and IGF-I-depleted mice measured by staining with the mitochondrial specific mitotracker green fluorescent dye as described in the Materials and Methods. **A.** Mean mitochondrial mass of the cell populations at passage 2 and 10 is presented as analyzed by flow cytometery. Differences between the mitochondrial mass in the IGF-I deficient mice and controls was significant (*P*<0.01 at passage 2 and *P*<0.05 at passage 10). **B.** Relative mitochondrial DNA content at passage 2 is presented. The experiment presented is representative of the results of 2 independent measurements on 4 DNA isolates using independent primer sets [45] that amplify mitochondrial and nuclear DNA. The difference in mitochondrial DNA content was significant (P<0.05).

In order to determine whether altering IGF-I levels in the whole organism results in similar changes in autophagy as those observed in culture, we examined mice that produce reduced levels of IGF-I. These mice harbor a hypomorphic allele of the *Igf1* gene due to an insertion in exon 3 [28]. We have confirmed that the mice produce 40–50% lower levels of IGF-I in both the serum and all tissues tested (kidney, brain, muscle liver, heart) (data not shown). We examined tissues from the IGF-I deficient mice for evidence of autophagy by staining for LC-3 puncta. We found increased numbers of LC-3 containing puncta in the tissues of the IGF-I deficient mice relative to controls (Fig 7a,b). In addition, we found increased LC3-I when total protein extracts are examined by Western blot analysis (Fig 7b). Thus, in the low IGF-I mice we find an increase in LC-3 levels combined with an increase in LC3 containing puncta suggesting an increase in autophagy, while in the cell cultures LC3 levels tended to be lower in conditions of increased autophagy, in serum free medium. This suggests that there are some differences in the dynamics of autophagy in vivo and in vitro but the consistent finding was that high IGF-I levels lead to reduced autophagy. In order to determine whether mitochondrial differences occur in cells derived from the low IGF-I environment present in the IGF-I deficient mice, we examined embryo fibroblast cells derived from either IGF-I deficient mice or controls. Mitochondrial mass was examined using mitotracker green, a fluorescent dye which preferentially localizes to the mitochondrial membrane and can be used to estimate mitochondrial mass. Flow cytometry analysis revealed that embryo fibroblasts derived from the IGF-I deficient mice had a significantly greater mitochondrial mass than fibroblasts derived from control mice. This difference was greatest at low passage and decreased with increasing passages in culture presumably as the cells became acclimatized to culture conditions and the influence of the altered hormone environment in the whole animal was lost (Fig 8A). Mitochondrial DNA copy number was also compared in the MEF cultures at passage 2. A qPCR analysis using primer sets targeting either the mitochondrial or nuclear genome indicated that MEFs derived from the IGF-I deficient mice contained a higher mitochondrial DNA content than the control MEFs (Fig 8B).

## Discussion

We provide evidence that autophagy occurs in quiescent cells even when sufficient nutrients are available and that inhibition of autophagy through IGF-I signaling can lead to the accumulation of cells with dysfunctional mitochondria and decreased long-term viability. Rapamycin, which enhances autophagy, can ameliorate the effects of IGF-I while inhibition of autophagy recapitulates some of these effects. Furthermore, it appears that a reduction in IGF-I in mice leads to enhanced autophagy and a similar decrease in depolarized mitochondria. Interestingly, this is accompanied by an increase in total mitochondrial mass.

In total, the results indicate that inhibition of autophagy by IGF-I decreases cell viability through interference with mitochondrial turnover. These observations suggest that increased IGF-I signaling over long periods have unanticipated consequences that are distinct from the pro survival effects observed in acute settings.

Autophagy has been identified as a process for the turnover of intracellular components which can be negatively regulated through the intracellular signaling pathway associated with mTOR [29]. It involves a series of lysine-linked conjugation steps analogous to the ubiquitin conjugation required for proteasome targeting [30]. This process is important for proper cellular function and defects in autophagy have been linked to several types of degenerative diseases [31]. Although direct evidence that autophagy can influence longevity in mammals is lacking, experimental evidence suggests that an enhanced rate of autophagy during aging enhances liver function [32] and appropriate levels of autophagy are essential for cardiac function [33]. Autophagy has been linked to aging, and a reduction in autophagy during aging has been observed in rodents and other organisms [34], [35]. Caloric restriction increases autophagy in rodents [36], and genetic studies in *Caenorhabditis elegans* indicate that autophagy may be required for life-span extension by caloric restriction [37]. Autophagy also increases during dauer formation in *C. elegans* and is required for life-span extension in daf-2 mutants [38]. Genetic studies in *C. elegans* have found that autophagy genes are required for life-span extension in response to mutations in the insulin/insulin-like growth factor (IGF) receptor [38] and to mutations that induce caloric restriction [37], although there may be caveats to this connection that have not been fully appreciated since other studies indicate that suppression of autophagy in the adult may extend life span [39]. Autophagy is an important mechanism for the clearance of mitochondria [40] following damage and IGF-I has been reported to influence this process [41] but the relative importance of mitochondrial clearance under physiologic conditions is less clear. Surprisingly, we find that even in the absence of starvation, autophagy may play an important role in the normal turnover of mitochondria and that inhibition of autophagy by IGF-I can lead to mitochondrial dysfunction and decreased cell viability. Because mitochondrial mutations and dysfunction may increase with age [42], [43], the reduced clearance of dysfunctional mitochondria in settings where IGF-I is elevated may be significant to age-related pathologic conditions.

## Materials and Methods

### Cell culture

All culture reagents were from Cellgro (Manassas, VA) unless otherwise stated. WI-38 human diploid fibroblasts were grown and in MEM supplemented with 10% fetal bovine serum, 1% L-glutamine, MEM non essential amino acids, MEM vitamins and 1% penicillin-streptomycin according to standard culture protocol [44]. For long term IGF-I stimulation experiments, cells were plated at 1×10^4^ cells/cm^2^ and placed in MCDB 105 (Sigma, St Louis, MO) with or without 40 ng/ml IGF-I (Gemini Biosciences, West Sacramento, CA) and/or 10 nM rapamycin (Enzo Biologicals, Plymouth Meeting, PA) for the indicated times after 24–48 hours. Culture medium was replaced every 3–4 days. Mouse Embryonic Fibroblasts (MEFs) from wild type and IGF-I deficient mice were isolated from E19 embryos by trypsin digestion and grown in MEM supplemented with 10% fetal bovine serum, 1% L-glutamine, MEM non essential amino acids, and 1% penicillin-streptomycin according to standard culture protocol [44]. All experiments with MEFs were performed on log phase cultures 48–72 hours after seeding at 1×10^4^/cm^2^.

### Clonal growth

For clonal growth assay, cells maintained in MCDB 105 medium for 2 weeks +/− IGF-I and/or rapamycin were harvested in 2.5% trypsin-EDTA, resuspended in full growth medium and 3×10^3^ or 3×10^4^ cells were seeded in 10 cm^2^ plates and cultured in full growth medium for 2 weeks. Plates were then rinsed with PBS and stained with a 0.05% crystal violet-50% methanol solution.

### Mitochondrial potential, mitochondrial mass, and mitochondrial DNA content

For mitochondrial potential studies, cells were incubated with 5 µg/ml JC-1 (Molecular Probes, Carlsbad, CA) at 37 C in 5% CO_2_ for 30 minutes, harvested in 2.5% trypsin-EDTA, resuspended in 200 µl full growth medium and analyzed immediately with a Guava Easy-Cyte Mini using the Guava Express Plus program. For mitochondrial mass evaluation, cells were incubated for 30 minutes in 100 nM Mitotracker Green FM (Molecular Probes, Carlsbad, CA) at 37 C, harvested in 2.5% trypsin-EDTA and resuspended in 200 µl full growth medium and analyzed immediately with a Guava-Easy-Cyte Mini using the Guava Express Plus program.

Relative mitochondrial DNA content was evaluated according to published methods [45]. Total DNA was extracted using phenol/chloroform extraction using the phase-lock gel system (5Prime Inc. Gaithersburg, MD) followed by ethanol precipitation and a second phenol/chloroform extraction. Primers used to amplify mitochondrial and nuclear DNA targeted the mitochondrial cytochrome C oxidase 1 subunit and the nuclear encoded NADH dehydrogenase [ubiquinone] flavoprotein 1 gene or a portion of the D loop in the mitochondrial genome and a noncoding region of the telomerase gene [45], [46].

### Western blotting

30 µg of protein extracts were run on SDS-PAGE and transferred onto Immobilon P PVDF membranes (Millipore, Billerica, MA). Blots were incubated with antibodies against LC3B, beta-Tubulin (Cell Signaling, Danvers, MA), p62 (Biomol, Plymouth Meeting, PA), beta-actin (Sigma, St Louis, MO), according to manufacturer's instructions.

### Fluorescence microscopy

Cells were plated at 10^4^ cells/cm^2^ on coverslips. For LC3-GFP live imaging, the coverslips were placed onto microscope slides and micrographs were acquired immediately. For immunofluorescence studies, cells were fixed in 4% paraformaldehyde, permeabilized in 0.25% Triton, incubated overnight with rabbit polyclonal anti-LC3B antibody (Cell Signaling, Danvers, MA), stained with goat anti-rabbit Alexa Fluor 488 conjugated (Invitrogen, Carlsbad, CA), counterstained with DAPI (Sigma, St Louis, MO) and the coverslips mounted on microscope slides with Vectashield mounting medium (Vector Laboratories, Burlingame, CA).

Paraffin embedded formalin fixed tissue slides from wild type and IGF-I-deficient mice were incubated overnight with rabbit polyclonal anti-LC3 B antibody (Cell Signaling, Danvers, MA), stained with a 1∶2 mixture or goat anti-rabbit/donkey anti-goat Alexa Fluor 488 conjugated (Invitrogen, Carlsbad, CA), counterstained with DAPI (Sigma, St Louis, MO) and mounted with Vectashield mounting medium (Vector Laboratories, Burlingame, CA). Images were acquired with an Olympus BX61 fluorescence microscope using the Slidebook 4 (version 4.0.1.44) software.

### Metabolic labeling

WI-38 cells were labeled in normal growth medium supplemented with 66 µCi/ml of ^35^S-Methionine (Perkin Elmer, Bridgeville, PA) for 30 hour, washed with PBS and chased in MCDB 105 with or without 40 ng/ml IGF-I for the indicated time points. Protein lysates were subject to tri-chloro-acetate precipitation and radioactivity was assessed through scintillation counting.

### Expression plasmids

The MigR1-GFP-LC3 retroviral plasmid was kindly provided by Dr. Mauricio Reginato (Drexel University COM). The Mission shRNA against ATG5 (cat: TRCN0000150645) was purchased from Sigma Aldrich, St Louis, MO. The scramble shRNA lentiviral plasmid was from Addgene, cat 1864, [24].

### RNA extraction and qRT-PCR

Total RNA from shATG5 infected cells was extracted 72 h post infection and qRT-PCR was performed using the Verso Sybr 1 step qRT kit (Thermo Scientific, Waltham, MA) in a Stratagene Mx3000P thermocycler.

### Statistical analysis

Unless otherwise stated in figure legend, all results are expressed as average plus or minus standard deviation of three independent samples. Significance was assessed with the unpaired, 2 tailed Student's t test.

### Ethics Statement

This study was carried out in accordance with the Guide for the Care and Use of Laboratory Animals of the National Institutes of Health under protocols approved by the IACUC committee of Drexel University.
